# *N*,*N*-Dibenzyl­methane­sulfonamide

**DOI:** 10.1107/S1600536808015055

**Published:** 2008-06-07

**Authors:** Mrityunjoy Datta, Alan J. Buglass, Chang Seop Hong, Jeon Hak Lim

**Affiliations:** a Department of Chemistry, Korea Advanced Institute of Science and Technology, Daejeon 305-701, Republic of Korea; b Department of Chemistry, Korea University, Seoul 136-701, Republic of Korea

## Abstract

Mol­ecules of the title compound, C_15_H_17_NO_2_S, which was synthesized from methane­sulfonyl chloride and dibenzyl­amine, are packed in anti­parallel arrays along the *c* axis, with the methyl group of one mol­ecule dovetailed between the two phenyl rings of the next mol­ecule. Along any such array, the sulfonyl O atoms protrude alternately up and down.

## Related literature

For crystallographic literature on sulfonamides such as methane­sulfonamides, see: Gowda *et al.* (2007 ▶). For literature on *N*,*N*-dialkyl­methane­sulfonamides, see: van Otterlo *et al.* (2004 ▶). For the synthesis, see: Banks & Hudson (1986 ▶); Stretter *et al.* (1969 ▶); Youn & Herrmann (1986 ▶).
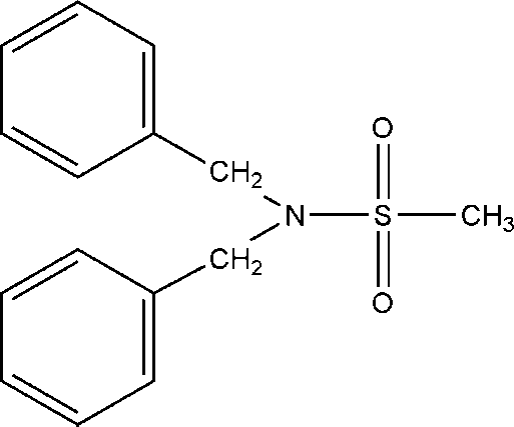


## Experimental

### 

#### Crystal data

C_15_H_17_NO_2_S
*M* *_r_* = 275.36
Orthorhombic, 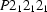

*a* = 6.0948 (1) Å
*b* = 13.4498 (4) Å
*c* = 17.1293 (4) Å
*V* = 1404.15 (6) Å^3^
*Z* = 4
Mo *K*α radiation
μ = 0.23 mm^−1^
*T* = 293 (2) K
0.32 × 0.10 × 0.08 mm

#### Data collection

Bruker APEXII diffractometer
Absorption correction: multi-scan (*SADABS*; Sheldrick, 1996 ▶) *T*_min_ = 0.931, *T*_max_ = 0.982
8426 measured reflections
3421 independent reflections
2694 reflections with *I* > 2σ(*I*)
*R*_int_ = 0.025

#### Refinement

*R*[*F* ^2^ > 2σ(*F* ^2^)] = 0.036
*wR*(*F* ^2^) = 0.095
*S* = 1.10
3421 reflections
173 parameters
H-atom parameters constrained
Δρ_max_ = 0.14 e Å^−3^
Δρ_min_ = −0.29 e Å^−3^
Absolute structure: Flack (1983 ▶), 1428 Friedel pairs
Flack parameter: 0.01 (8)

### 

Data collection: *APEX2* (Bruker, 2001 ▶); cell refinement: *SAINT* (Bruker, 2001 ▶); data reduction: *SAINT*; program(s) used to solve structure: *SHELXTL* (Sheldrick, 2008 ▶); program(s) used to refine structure: *SHELXL97* (Sheldrick, 2008 ▶); molecular graphics: *ORTEP-3* (Farrugia, 1997 ▶); software used to prepare material for publication: *WinGX* (Farrugia, 1999 ▶).

## Supplementary Material

Crystal structure: contains datablocks I, global. DOI: 10.1107/S1600536808015055/ng2454sup1.cif

Structure factors: contains datablocks I. DOI: 10.1107/S1600536808015055/ng2454Isup2.hkl

Additional supplementary materials:  crystallographic information; 3D view; checkCIF report

## Figures and Tables

**Table 1:** Hydrogen-bond geometry (Å, °)

*D*—H⋯*A*	*D*—H	H⋯*A*	*D*⋯*A*	*D*—H⋯*A*
C14—H14*B*⋯O1^i^	0.97	2.50	3.366 (2)	149
C7—H7*B*⋯O1	0.97	2.44	2.911 (2)	109
C8—H8⋯N1	0.93	2.61	2.937 (2)	101

Symmetry code: (i) 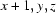.

**Fig. 1.**
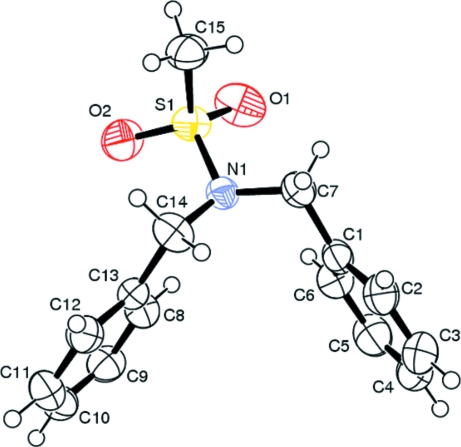
Molecular structure of (I), with atom labels and 50% probability displacement ellipsoids for non-H atoms.

**Fig. 2.**
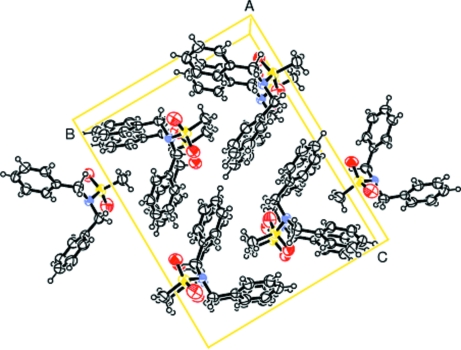
The packing of (I), viewed down the a axis.

## supplementary crystallographic information

### Comment

The title compound (I) was prepared by an established method (Stretter *et al.*, 1969) from dibenzylamine and methanesulfonyl chloride. The last-named compound was prepared unintentionally from methyl sulfide, sulfuryl chloride and acetic acid (Youn and Herrmann, 1986), but with an excess of sulfuryl chloride (1:4:2). With the normal ratio of reactants (1:3:2), methanesulfinyl chloride is the main product. No observation of this kind was reported by the original authors, and indeed, oxidation of disulfides or sulfinyl moeities to sulfonyl moeities generally needs the use of peroxyacids or hydroperoxides.

The molecular structure of (I) (Fig. 1) exhibits no unusual bond lengths or bond angles. The crystal packing of (I) (Fig. 2) shows antiparallel arrays along the *c* axis, with the *S*-methyl group occupying the space between the two benzene rings of the next molecule. Along each of these arrays, the oxygen atoms point alternately up and down, and there appears to be some stacking of benzene rings between molecules, along the a and b axes. There is no evidence of hydrogen bonding, but there are weak C14—H14···O1, C7—H7···O2 and C8—H8···N1 interactions (Table 1).

### Experimental

Methanesulfonyl chloride was prepared by the method of Youn and Herrmann, but using an excess of sulfuryl chloride (*viz*. methyl disulfide (0.01 mol), acetic acid (0.02 mol) and sulfuryl chloride (0.04 mol)). The title compound was prepared by the method of Stretter *et al.*, using dibenzylamine (1.5 g, 8 mmol), methanesulfonyl chloride (458 mg, 4 mmol) and dichloromethane (30 ml). The crude product was purified by column chromatography on silica gel using dichloromethane as eluent, giving *N*,*N*-dibenzylmethanesulfonamide as white crystals (1.05 g, 96%), mp, 83–85°C. Literature mp. 84–85 *^o^*C (Banks & Hudson, 1986).

Crystals were obtained by evaporation of solvent from a solution of (I) in dichoromethane/hexane (1:4).

FTIR (KBr) (cm^-1^) 3088, 3062, 3009, 1496, 1446, 1438, 1382, 1318, 1265, 1207, 1132, 1091, 1056, 949

^1^H NMR (400 MHz, CDCl_3_, p.p.m. with respect to TMS) 7.39–7.29 (m, 10H), 4.35 (s, 4H), 2.77 (s, 3H)

^13^C NMR (100 MHz, CDCl_3_, p.p.m. with respect to TMS) 135.4, 128.7, 128.0, 49.8, 40.2

EIMS m/*z* (%) 275 (*M*^+.^, 19), 196 (*M*^+.^ - CH_3_SO_2_^.^, 84), 195 (82), 184 (84), 91 (100)

Anal. Calcd. for C_15_H_17_NO_2_S (%): C, 65.45; H, 6.18; N, 5.09; S, 11.63. Found (%): C, 65.22; H, 6.15; N, 5.03; S, 11.80.

### Refinement

H atoms were located on a difference Fourier map, positioned geometrically and refined using a riding model, with C—H = 0.93–0.97 Å and with *U*_iso_(H) = 1.2 (1.5 for CH_3_) times *U*_eq_(C).

### Crystal data

**Table:**

C_15_H_17_NO_2_S	*F*_000_ = 584
*M**_r_* = 275.36	*D*_x_ = 1.303 Mg m^−^^3^
Orthorhombic, *P*2_1_2_1_2_1_	Mo *K*α radiation λ = 0.71073 Å
Hall symbol: P 2ac 2ab	Cell parameters from 3097 reflections
*a* = 6.0948 (1) Å	θ = 3.0–26.3º
*b* = 13.4498 (4) Å	µ = 0.23 mm^−^^1^
*c* = 17.1293 (4) Å	*T* = 293 (2) K
*V* = 1404.15 (6) Å^3^	Plate, white
*Z* = 4	0.32 × 0.10 × 0.08 mm

### Data collection

**Table:**

Bruker APEXII diffractometer	3421 independent reflections
Radiation source: fine-focus sealed tube	2694 reflections with *I* > 2σ(*I*)
Monochromator: graphite	*R*_int_ = 0.025
*T* = 293(2) K	θ_max_ = 28.3º
ω scans	θ_min_ = 1.9º
Absorption correction: multi-scan(SADABS; Sheldrick, 1996)	*h* = −8→7
*T*_min_ = 0.931, *T*_max_ = 0.982	*k* = −17→15
8426 measured reflections	*l* = −18→22

### Refinement

**Table:**

Refinement on *F*^2^	Hydrogen site location: inferred from neighbouring sites
Least-squares matrix: full	H-atom parameters constrained
*R*[*F*^2^ > 2σ(*F*^2^)] = 0.036	*w* = 1/[σ^2^(*F*_o_^2^) + (0.0517*P*)^2^] where *P* = (*F*_o_^2^ + 2*F*_c_^2^)/3
*wR*(*F*^2^) = 0.095	(Δ/σ)_max_ = 0.001
*S* = 1.10	Δρ_max_ = 0.14 e Å^−^^3^
3421 reflections	Δρ_min_ = −0.29 e Å^−^^3^
173 parameters	Extinction correction: none
Primary atom site location: structure-invariant direct methods	Absolute structure: Flack (1983), 1428 Friedel pairs
Secondary atom site location: difference Fourier map	Flack parameter: 0.01 (8)

### Special details

**Table:**

Geometry. All e.s.d.'s (except the e.s.d. in the dihedral angle between two l.s. planes) are estimated using the full covariance matrix. The cell e.s.d.'s are taken into account individually in the estimation of e.s.d.'s in distances, angles and torsion angles; correlations between e.s.d.'s in cell parameters are only used when they are defined by crystal symmetry. An approximate (isotropic) treatment of cell e.s.d.'s is used for estimating e.s.d.'s involving l.s. planes.
Refinement. Refinement of *F*^2^ against ALL reflections. The weighted *R*-factor *wR* and goodness of fit *S* are based on *F*^2^, conventional *R*-factors *R* are based on *F*, with *F* set to zero for negative *F*^2^. The threshold expression of *F*^2^ > σ(*F*^2^) is used only for calculating *R*-factors(gt) *etc*. and is not relevant to the choice of reflections for refinement. *R*-factors based on *F*^2^ are statistically about twice as large as those based on *F*, and *R*- factors based on ALL data will be even larger.

### Fractional atomic coordinates and isotropic or equivalent isotropic displacement parameters (Å^2^)

**Table:**

	*x*	*y*	*z*	*U*_iso_*/*U*_eq_	
C1	0.6426 (3)	0.26792 (15)	0.41616 (10)	0.0536 (4)	
H1	0.7540	0.2208	0.4192	0.064*	
C2	0.6736 (4)	0.36030 (17)	0.44782 (11)	0.0659 (6)	
H2	0.8057	0.3754	0.4723	0.079*	
C3	0.5109 (4)	0.43099 (17)	0.44367 (11)	0.0697 (6)	
H3	0.5322	0.4935	0.4656	0.084*	
C4	0.3181 (4)	0.40880 (15)	0.40713 (12)	0.0645 (6)	
H4	0.2082	0.4566	0.4036	0.077*	
C5	0.2856 (3)	0.31565 (14)	0.37533 (11)	0.0518 (5)	
H5	0.1535	0.3011	0.3507	0.062*	
C6	0.4471 (3)	0.24390 (13)	0.37967 (9)	0.0437 (4)	
C7	0.4079 (3)	0.14042 (14)	0.34963 (10)	0.0515 (5)	
H7A	0.5423	0.1022	0.3542	0.062*	
H7B	0.2971	0.1084	0.3816	0.062*	
C8	0.3166 (3)	0.30315 (13)	0.15342 (10)	0.0476 (4)	
H8	0.2041	0.2963	0.1897	0.057*	
C9	0.3116 (4)	0.38057 (14)	0.09996 (11)	0.0551 (5)	
H9	0.1967	0.4260	0.1010	0.066*	
C10	0.4744 (4)	0.39038 (15)	0.04575 (11)	0.0614 (5)	
H10	0.4698	0.4422	0.0099	0.074*	
C11	0.6452 (4)	0.32373 (16)	0.04415 (11)	0.0626 (5)	
H11	0.7554	0.3301	0.0069	0.075*	
C12	0.6529 (3)	0.24733 (14)	0.09789 (10)	0.0501 (4)	
H12	0.7698	0.2029	0.0972	0.060*	
C13	0.4884 (3)	0.23622 (12)	0.15276 (9)	0.0381 (4)	
C14	0.5104 (3)	0.15067 (15)	0.20925 (10)	0.0508 (4)	
H14A	0.5184	0.0895	0.1793	0.061*	
H14B	0.6485	0.1579	0.2368	0.061*	
N1	0.3353 (2)	0.14007 (10)	0.26726 (7)	0.0411 (3)	
S1	0.13533 (6)	0.06492 (3)	0.24587 (3)	0.04857 (14)	
O1	−0.0312 (2)	0.07909 (13)	0.30334 (10)	0.0755 (4)	
O2	0.0845 (2)	0.08028 (11)	0.16558 (8)	0.0706 (4)	
C15	0.2358 (4)	−0.05725 (14)	0.25679 (12)	0.0625 (5)	
H15A	0.1233	−0.1038	0.2426	0.094*	
H15B	0.2779	−0.0679	0.3101	0.094*	
H15C	0.3610	−0.0665	0.2235	0.094*	

### Atomic displacement parameters (Å^2^)

**Table:**

	*U*^11^	*U*^22^	*U*^33^	*U*^12^	*U*^13^	*U*^23^
C1	0.0547 (10)	0.0613 (12)	0.0448 (10)	0.0043 (11)	−0.0069 (9)	0.0037 (9)
C2	0.0788 (14)	0.0725 (15)	0.0466 (11)	−0.0145 (13)	−0.0131 (10)	−0.0001 (10)
C3	0.1070 (17)	0.0532 (12)	0.0490 (12)	−0.0108 (15)	0.0061 (12)	−0.0087 (10)
C4	0.0838 (15)	0.0532 (12)	0.0565 (13)	0.0162 (12)	0.0131 (11)	−0.0011 (10)
C5	0.0494 (9)	0.0554 (12)	0.0506 (11)	0.0045 (9)	0.0012 (8)	0.0007 (9)
C6	0.0517 (9)	0.0427 (10)	0.0368 (9)	−0.0001 (8)	−0.0009 (7)	0.0050 (8)
C7	0.0658 (11)	0.0457 (10)	0.0431 (10)	0.0025 (9)	−0.0100 (8)	0.0083 (8)
C8	0.0486 (9)	0.0484 (10)	0.0459 (10)	0.0070 (8)	0.0010 (7)	0.0033 (8)
C9	0.0626 (12)	0.0447 (10)	0.0580 (12)	0.0091 (9)	−0.0101 (9)	0.0042 (9)
C10	0.0896 (14)	0.0486 (12)	0.0459 (11)	−0.0099 (11)	−0.0092 (11)	0.0095 (9)
C11	0.0761 (12)	0.0645 (13)	0.0472 (11)	−0.0042 (12)	0.0139 (10)	0.0025 (9)
C12	0.0519 (10)	0.0506 (10)	0.0478 (10)	0.0005 (10)	0.0077 (8)	−0.0032 (8)
C13	0.0413 (7)	0.0378 (9)	0.0352 (8)	−0.0007 (7)	−0.0025 (6)	−0.0018 (7)
C14	0.0419 (9)	0.0528 (11)	0.0577 (11)	0.0103 (8)	0.0060 (8)	0.0116 (9)
N1	0.0426 (7)	0.0420 (8)	0.0387 (7)	0.0012 (6)	−0.0023 (6)	0.0043 (6)
S1	0.0398 (2)	0.0481 (2)	0.0578 (3)	0.00017 (19)	−0.0094 (2)	0.0060 (2)
O1	0.0430 (7)	0.0847 (11)	0.0987 (11)	0.0038 (8)	0.0144 (7)	0.0118 (9)
O2	0.0796 (9)	0.0658 (9)	0.0665 (9)	−0.0051 (8)	−0.0352 (7)	0.0106 (7)
C15	0.0804 (12)	0.0414 (10)	0.0655 (13)	−0.0019 (10)	−0.0118 (11)	0.0052 (10)

### Geometric parameters (Å, °)

**Table:**

C1—C2	1.369 (3)	C9—H9	0.930
C1—C6	1.384 (3)	C10—C11	1.374 (3)
C1—H1	0.930	C10—H10	0.930
C2—C3	1.376 (3)	C11—C12	1.380 (3)
C2—H2	0.930	C11—H11	0.930
C3—C4	1.364 (3)	C12—C13	1.382 (2)
C3—H3	0.930	C12—H12	0.930
C4—C5	1.380 (3)	C13—C14	1.509 (2)
C4—H4	0.930	C14—N1	1.465 (2)
C5—C6	1.380 (2)	C14—H14A	0.970
C5—H5	0.930	C14—H14B	0.970
C6—C7	1.503 (3)	N1—S1	1.6250 (14)
C7—N1	1.479 (2)	S1—O2	1.4247 (13)
C7—H7A	0.970	S1—O1	1.4269 (14)
C7—H7B	0.970	S1—C15	1.764 (2)
C8—C13	1.381 (2)	C15—H15A	0.960
C8—C9	1.387 (3)	C15—H15B	0.960
C8—H8	0.9300	C15—H15C	0.960
C9—C10	1.366 (3)		
			
C2—C1—C6	120.7 (2)	C11—C10—H10	120.0
C2—C1—H1	119.7	C10—C11—C12	119.9 (2)
C6—C1—H1	119.7	C10—C11—H11	120.1
C1—C2—C3	120.5 (2)	C12—C11—H11	120.1
C1—C2—H2	119.8	C11—C12—C13	120.61 (19)
C3—C2—H2	119.8	C11—C12—H12	119.7
C4—C3—C2	119.6 (2)	C13—C12—H12	119.7
C4—C3—H3	120.2	C8—C13—C12	119.02 (15)
C2—C3—H3	120.2	C8—C13—C14	124.00 (15)
C3—C4—C5	120.2 (2)	C12—C13—C14	116.98 (15)
C3—C4—H4	119.9	N1—C14—C13	116.36 (14)
C5—C4—H4	119.9	N1—C14—H14A	108.2
C6—C5—C4	120.7 (2)	C13—C14—H14A	108.2
C6—C5—H5	119.6	N1—C14—H14B	108.2
C4—C5—H5	119.6	C13—C14—H14B	108.2
C5—C6—C1	118.34 (17)	H14A—C14—H14B	107.4
C5—C6—C7	121.06 (16)	C14—N1—C7	115.38 (14)
C1—C6—C7	120.50 (16)	C14—N1—S1	116.99 (12)
N1—C7—C6	112.15 (14)	C7—N1—S1	116.21 (11)
N1—C7—H7A	109.2	O2—S1—O1	119.46 (10)
C6—C7—H7A	109.2	O2—S1—N1	106.89 (8)
N1—C7—H7B	109.2	O1—S1—N1	107.15 (8)
C6—C7—H7B	109.2	O2—S1—C15	108.24 (10)
H7A—C7—H7B	107.9	O1—S1—C15	107.36 (10)
C13—C8—C9	120.04 (17)	N1—S1—C15	107.16 (9)
C13—C8—H8	120.0	S1—C15—H15A	109.5
C9—C8—H8	120.0	S1—C15—H15B	109.5
C10—C9—C8	120.37 (19)	H15A—C15—H15B	109.5
C10—C9—H9	119.8	S1—C15—H15C	109.5
C8—C9—H9	119.8	H15A—C15—H15C	109.5
C9—C10—C11	120.07 (18)	H15B—C15—H15C	109.5
C9—C10—H10	120.0		

### Hydrogen-bond geometry (Å, °)

**Table:**

*D*—H···*A*	*D*—H	H···*A*	*D*···*A*	*D*—H···*A*
C14—H14B···O1^i^	0.97	2.50	3.366 (2)	149
C7—H7B···O1	0.97	2.44	2.911 (2)	109
C8—H8···N1	0.93	2.61	2.937 (2)	101

Symmetry codes: (i) *x*+1, *y*, *z*.
